# Analysis and simulation study of the HIV/AIDS model using the real cases

**DOI:** 10.1371/journal.pone.0304735

**Published:** 2024-06-25

**Authors:** Mutum Zico Meetei, Mahmoud H. DarAssi, Muhammad Altaf Khan, Ali N. A. Koam, Ebraheem Alzahrani, Abdullah Ali H. Ahmadini

**Affiliations:** 1 Department of Mathematics, College of Science, Jazan University, Jazan, Kingdom of Saudi Arabia; 2 Department of Basic Sciences, Princess Sumaya University for Technology, Amman, Jordan; 3 Faculty of Natural and Agricultural Sciences, University of the Free State, Bloemfontein, South Africa; 4 Department of Mathematics, Faculty of Science, King Abdulaziz University, Jeddah, Saudi Arabia; Lahore School of Economics, PAKISTAN

## Abstract

We construct a model to investigate HIV/AIDS dynamics in real cases and study its mathematical analysis. The study examines the qualitative outcomes and confirms the local and global asymptotic stability of both the endemic equilibrium and the disease-free equilibrium. The model’s criteria for exhibiting both local and global asymptotically stable behavior are examined. We compute the endemic equilibria and obtain the existence of a unique positive endemic equilibrium. The data is fitted to the model using the idea of nonlinear least-squares fitting. Accurate parameter values are achieved by fitting the data to the model using a 95% confidence interval. The basic reproduction number is computed using parameters that have been fitted or estimated. Sensitivity analysis is performed to discover the influential parameters that impact the reproduction number and the eradication of the disease. The results show that implementing preventive measures can reduce HIV/AIDS cases.

## 1 Introduction

One of the biggest threats to global health and development is HIV, which is the cause of AIDS. HIV attacks white blood cells in the human body, impairing the immune system. AIDS may originate from an HIV infection and might progress into an extremely serious health issue. AIDS is an infectious disease that causes impaired immune system functionality. HIV is responsible for the impairment of the body’s ability to combat infections. In 1981, the first AIDS infection was identified, and the disease rapidly spread around the world. Globally, about 2 million individuals perished in 2009, and the illness is still expanding [1]. As a result, more stringent antiretroviral therapy is needed to minimize mortality [2, 3]. According to the findings, initiating antiretroviral therapy (ART) earlier in the course of HIV infection, when the body’s defense mechanism is stronger, leads to improved health outcomes over the long run [4, 5].

Because of the lack of a vaccine, various challenges are to treating infected people with AIDS. Antiretroviral therapy for/AIDS now includes taking two or more antiviral medications at the same time, typically from the protease and reverse transcriptase inhibitor (RTIs) classes (PIs) [6, 7]. The World Health Organization has made significant efforts to enhance antiretroviral therapy and the health care system, with the most prevalent treatment being highly active antiretroviral therapy (HAART) [8]. HIV-positive people should seek treatment before acquiring AIDS to limit the risk of transmission to others. In 1997, less than 300 infants in the US were infected with HIV/AIDS through vertical transmission, despite data on mother-to-child transmission suggesting that roughly 40% of cases are mother-to-child transmission [9, 10]. Approximately 2.5 million children younger than 15 years old died in Sub-Saharan Africa due to AIDS during the pregnancies of their mothers. Most of these kids were breastfed or otherwise exposed to HIV.

The virus continues to spread in all countries globally, and numerous nations have witnessed a rise in new infections after a previous decrease. High rates of HIV infection can be seen in several countries with a majority of Muslims, such as those in North Africa, the Middle East, and some parts of Asia [11]. However, the prevalence of HIV infections in Middle Eastern nations is quite low, mostly owing to many factors, including societal and cultural taboos [12]. In 2004, the MOH in Saudi Arabia initiated two nationwide screening programs. Premarital screening was one of these programs that was made mandatory in 2008 to help with the challenges of collecting data for incidences of infection [13]. As of 2019, the global population of individuals living with HIV/AIDS stood at 38 million, with an estimated annual occurrence of two million new HIV infections [14]. An estimated 6,30,000 people are expected to have died from HIV-related causes in 2022.

Pakistan reported its first incidence of HIV/AIDS in 1987, and since then, the number of cases has been rising [15]. This makes it easier to get illnesses such as TB, infections, and certain malignancies. In Pakistan, the HIV pandemic has spread mostly among the people who use the drug. In addition, male, female, and transgender sex workers (MSW, FSW, and TSW), along with migrant workers, are affected by the country’s significant drug usage and a lack of awareness of the prevalence of non-marital sexual activity in society [16]. One of the reasons for male, female, or transgender sex workers is poverty, and also they cannot afford the cost of marriage, except transgenders. In Pakistan, transgender people has a lot of issues, such as the registration for a Computerized National Identity Card (CNIC), where the government has now implemented some rules to require them to provide CNIC based on their status. This issue will solve many problems for transgender people trying to get government jobs, and due to this their involvement in sexual activities will be minimal.

Several studies have examined HIV/AIDS dynamics and causes. The authors considered the HIV/AIDS disease to study the disease and its control [17]. They used the concept that modifying antiviral sexual behaviors might aid disease transmission and antiviral (ARV) medicine as a kind of disease control. The authors considered the slow and rapid compartments of latent individuals for modeling HIV/AIDS [18]. They mentioned that for slow and rapid latent individuals the therapy is important. Mathematical modeling to study HIV/AIDS dynamics under the treatment of the HIV infected and AIDS individuals treatment has been studied in [19]. Using the HIV infection model with two delays are studied in [20]. Modeling HIV with weak CD4+ T cells has been explored in [21]. A non-integer model to analyze HIV/AIDS disease is considered in [22]. In [23], the influence of the fusion effect on HIV/AIDS modeling has been examined. Stability analysis associated with the HIV/AIDS disease model is discussed in [24]. The authors considered the reported cases of HIV/AIDS and presented a1989–2019 and compartmental model to explore their dynamics [25]. The concept of awareness and unawareness has been considered in the modeling and the results. The authors in [26] constructed a compartmental model by extending the concept of awareness and unawareness used in [25] for HIV/AIDS with optimal control analysis under the reported data of Ethiopia. In [27], the sexual and ART have been considered. They considered the real data for the period 1989–2019 and obtained the results. HIV modeling has been studied about the roles played by commercial sex workers and injecting drug users [28]. The HIV model with stochastic analysis is given in [29]. According to the authors in [30], numerical simulations showed that pneumonia vaccination and treatment against disease have the effect of decreasing pneumonia and coepidemic disease expansion, as well as reducing the progression rate of HIV infection to the AIDS stage. The authors in [31] investigated the effect of model parameter values on the number of reproductions for HIV/AIDS disease by simulating various scenarios numerically and identified the most sensitive parameters. By applying the Castillo-Chavez criteria, the authors of [32] demonstrated that the disease-free equilibrium is asymptotically stable on a global scale whenever the associated reproduction number is less than one. Recently, the Castillo-Chavez criteria were again used by the authors of [33] to demonstrate the global stability of the models’ disease-free equilibrium points. In order to ascertain the presence of co-infection between HIV/AIDS and pneumonia, the Centre multivariate criteria were utilized. In [34], the authors examined the most effective effects of four time-dependent control techniques on the transmission of the HBV and COVID-19 co-epidemic using a compartmental modeling approach.

UNAIDS statistics indicate that around 97,000 individuals in Pakistan were HIV-positive by 2009. Reported cases, as documented by official sources, are significantly smaller in number. Underreporting is widespread in many nations due to the social stigma associated with HIV, inadequate surveillance, voluntary counseling and testing programs, and a lack of awareness among the general public and medical professionals. Even though HIV prevalence is typically low, HIV is well-established among injecting drug users, and an epidemic among transgender individuals (6%) is spreading in some places. Female sex workers continue to have a low HIV prevalence. Given the protective benefits of circumcision, an epidemic of sexually transmitted diseases is improbable, but the high links between sex work and injecting drug use raise the risk that the disease may spread well beyond these populations. Injecting drug users (IDUs), HIV between male sex workers and transgenders, risky sexual practices of female sex workers, blood transfusion, migrants and refugees, and other factors all contribute to a rise in HIV incidence in the nation. The risk factors stated below are the most likely manner in which HIV is spreading rapidly in the nation [35].

There are various techniques available in the literature that solve mathematical models and other related problems arising in science and engineering, see [36–40]. Here, we give some details on the above-mentioned techniques and their applications to the solution of the problems. The authors in [36] used sine-Gordon expansion (SGE) and generalized Kudryashov (GK) schemes to generate broad-spectral solutions with unknown parameters, while in [37], studied the optical soliton solutions of a nonlinear Schrodinger equation involving the parabolic law of non-linearity. A fractional iteration algorithm is used in [38] for numerically solving nonlinear noninteger order partial differential equations, while in [39], the authors presented solutions to the wave-like vibration equations using the variational iteration algorithm-I. The variational iteration algorithm II is presented in [40] for solving the diffusion and convection-diffusion equations, giving accurate results, having a fast convergence rate, and having superior robustness when compared to alternative methods.

The authors in [41] used a new modified variational iteration algorithm-1 to numerically solve the coupled Burgers’ equations, providing extremely precise solutions. A similar approach is applied in [42] to accelerate the fast convergence of series solutions and further utilize KdV, mKdV, and combined KdV-mKdV equations to illustrate its reliability and accuracy. The authors in [43] used an efficient local meshless method for solving numerically the time fractional-order multi-dimensional diffusion PDE, while in [44] a local meshless collocation method based on mutoquadric radial basis function is used to numerically simulate the time-fractional Black-Scholes model. Two-term time-fractional PDE models were numerically solved utilizing an efficient and accurate local meshless technique in [45]. In [46], a well-known simulation method that used a modified (*G*′/*G*) method for the nonlinear predator-prey (NPP) system was updated to create hyperbolic, rational, and trigonometric numerical solutions.

We mentioned above the comprehensive detail of the literature on the mathematical models of HIV/AIDS. Based on the above literature, no one mentioned the HIV/AIDS model using the reported cases from Pakistan and their solution. This work will analyze the model using the reported cases of HIV/AIDS in Pakistan and provide details on how the cases in Pakistan can be minimized. The purpose of this work is to use Pakistan’s reported HIV/AIDS statistics from 1992 to 2020 to build a mathematical model. The model includes the treatment of HIV-infected people and susceptible people who alter their sexual practices per unit period, the impact of treatment failure, and the predictions of the model parameters in the long-run disease in Pakistan from 1992. Later, we use the real data year-wise from Pakistan from 1992 to 2020 and obtain the fitting to the data. The simulation result regarding disease spread and control is shown.

The following part of the paper has been divided into subsequent sections: Section 2 provides a detailed explanation of the model design, analysis, and fundamental findings. Section 3 explores the qualitative study of the equilibrium points associated with the model. Using the data fitting to the model and determining sensitive parameters to perform sensitivity analysis have been discussed in detail in Section 4. Numerical results regarding the model and its discussion are provided in Section 5. Lastly, the results are briefly outlined in Section 6.

## 2 HIV/AIDS model framework

We propose a mathematical model for HIV/AIDS to investigate the disease dynamics using the reported data. To formulate the model, we represent the total population, indicated by *N*(*t*), and split it into five different subclasses: Individuals that are healthy but have the risk of infection, given by *S*(*t*), people that are infected with HIV, are known to be HIV-infected, *I*(*t*), individuals that have full-blown AIDS but do not receive ARV treatment are given by *A*(*t*); people that are treated, *T*(*t*), people who have changed their sexual behaviors sufficiently to be immune to the spread of HIV through sexual activity, are denoted as *R*(*t*). So, we have

$$\begin{array}{c} N(t)=S(t)+I(t)+A(t)+T(t)+R(t). \end{array}$$

The population of healthy individuals is created through the natural birth rate Π, and reduced by the natural death rate *ρ*. Healthy individuals get an infection while in contact with the infected individuals, through the transmission route given by

$$\begin{array}{c} \Psi =\tau \frac{\left(I+\beta_{1}T+\beta_{2}A\right)}{N}, \end{array}$$

where *τ* is the effective HIV infection contact rate. The parameter *β*_1_ denotes the partial recovery of immune function in HIV-infected persons who utilize ART appropriately. *β*_2_ is the modification parameter that determines the relative transmission of people having AIDS symptoms compared to those who are HIV positive but do not exhibit AIDS symptoms.

This effective rate, Ψ, and *ω*, the rate at which healthy people alter their sexual practices per unit period, decrease the susceptible population. The above discussion can be shown using the nonlinear ordinary differential equations,

$$\begin{array}{c} \frac{dS}{dt}=\Pi -\Psi S-\left(\omega +\rho \right)S. \end{array}$$ (1)

The population of HIV-infected people is generated through the effective contact rate Ψ, and people in the treatment class at a rate *ν*_1_. It is decreased through the natural death rate *ρ*, individuals acquire an AIDS transfer rate of *ψ*_1_, and those at a rate *ψ*_2_ join the treatment class. The rate of change is provided by

$$\begin{array}{c} \frac{dI}{dt}=\Psi S+\nu_{1}T-\left(\rho +\psi_{1}+\psi_{2}\right)I \end{array}$$ (2)

The populations of the AIDS population generated through the transfer rate *ψ*_1_, and those who have failed in treatment have a rate *ν*_2_. It is reduced by the natural and disease-related death rates *ρ*, and *d*_1_ respectively. We give the below equation for the above discussion,

$$\begin{array}{c} \frac{dA}{dt}=\psi_{1}I-\left(d_{1}+\rho \right)A+\nu_{2}T. \end{array}$$ (3)

The treatment class is created using the transfer rate *ψ*_2_, and it is lowered by the natural death rate *ρ*, disease death rate *d*_2_, individuals joining back the HIV-infected population *ν*_1_, and those in treatment failure *ν*_2_. This process is given by

$$\begin{array}{c} \frac{dT}{dt}=\psi_{2}I-\left(\rho +d_{2}+\nu_{1}+\nu_{2}\right)T. \end{array}$$ (4)

The population of people resistant to HIV is increased only through the rate *ω* while it is reduced by the natural death rate *ρ*, and hence the mathematical representation is given by

$$\begin{array}{c} \frac{dR}{dt}=\omega S-\rho R. \end{array}$$ (5)

Individuals who follow treatment procedures have a low chance of developing AIDS-associated symptoms. The assumptions of the rate *ω* were previously studied for the HIV/AIDS model, and readers are referred to [17, 19] for additional information on this subject. The equations given in (1–5) are shown as a joint system, given by

$$\begin{array}{c} \left\{ \begin{array}{c} \frac{dS}{dt}=\Pi -\Psi S-\left(\omega +\rho \right)S,\\ \frac{dI}{dt}=\Psi S+\nu_{1}T-\left(\rho +\psi_{1}+\psi_{2}\right)I,\\ \frac{dA}{dt}=\psi_{1}I-\left(d_{1}+\rho \right)A+\nu_{2}T,\\ \frac{dT}{dt}=\psi_{2}I-\left(\rho +d_{2}+\nu_{1}+\nu_{2}\right)T,\\ \frac{dR}{dt}=\omega S-\rho R, \end{array}\right. \end{array}$$ (6)

with the initial conditions (ICs),

$$\begin{array}{c} S\left(0\right)=S_{0}\geq 0,\;I\left(0\right)=I_{0}\geq 0,\;A\left(0\right)=A_{0}\geq 0,\;T\left(0\right)=T_{0}\geq 0,\;R\left(0\right)=R_{0}\geq 0. \end{array}$$ (7)

**Fig 1:**
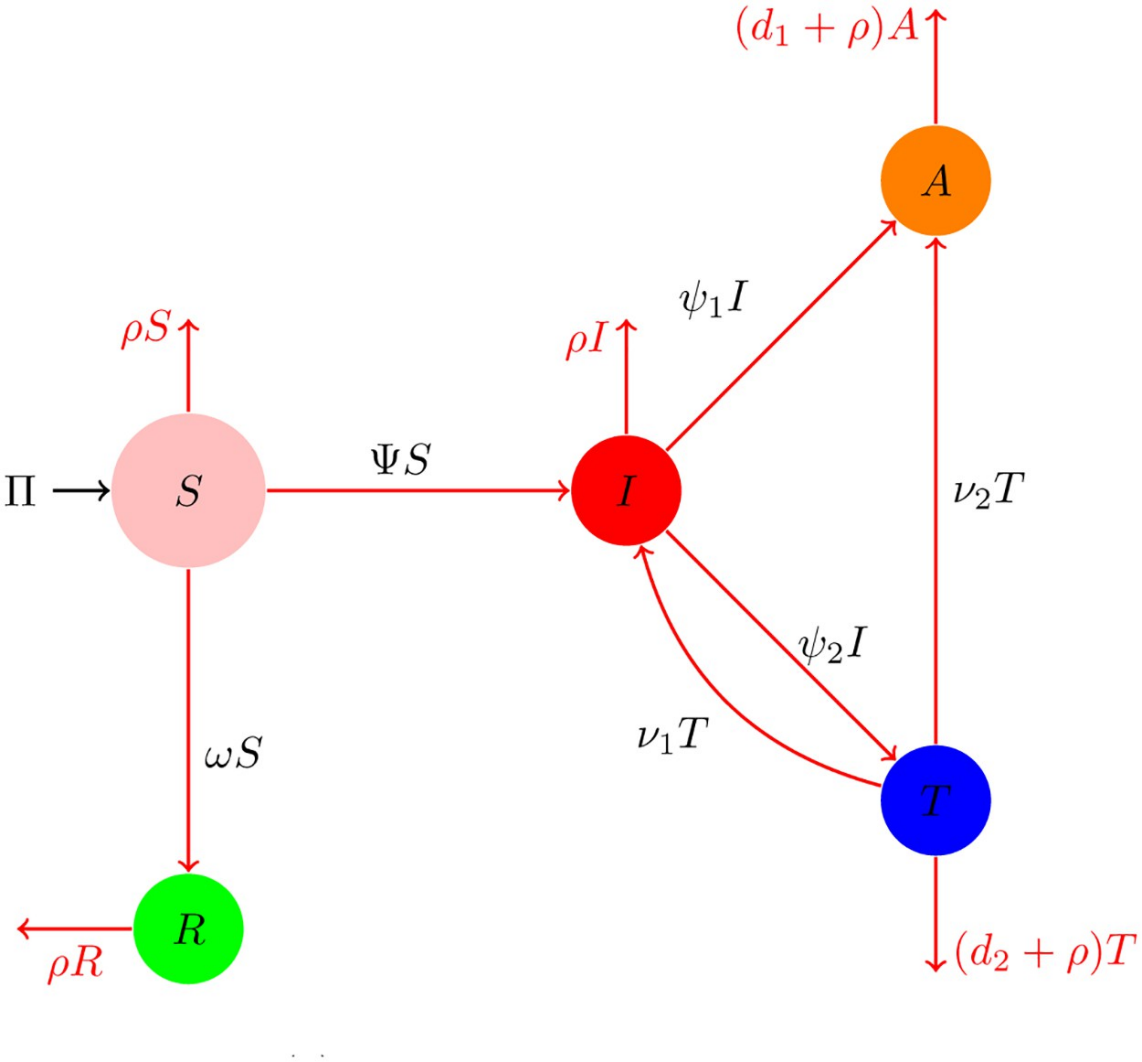
Rate of flow of the system (6).

Fig 1 represents the rate of flow of parameters from one state to another of the model (6). The definitions of the state variables and parameter details are given, respectively, in Tables 1 and 2.

**Table 1:** Details of variables involved in the model.

Notation	Details
*S*(*t*)	Susceptible individuals
*I*(*t*)	Infected with HIV
*A*(*t*)	Infected with AIDS
*T*(*t*)	People that are treated
*R*(*t*)	People that resist to HIV

**Table 2:** Details of parameters.

Notation	Details
Π	Birth rate
*ρ*	Natural death rate
*β*_1_	Modification parameter
*β*_2_	Modification parameter
*τ*	Effective contact rate
*ω*	Healthy people follow safe sexual practices
*ν*_1_	People joining infected class
*ν*_2_	Treatment failure
*ψ*_1_	The rate by which people join class *A*
*ψ*_2_	People that need to be treated
*d*_1_	Death rate in *T*
*d*_1_	Death rate in *A*

### 2.1 Model analysis

We have the total dynamics of the HIV/AIDS population, shown by

$$\begin{array}{ccc} \frac{dN}{dt} & = & \Pi -\rho N-d_{1}A-d_{2}T,\\ \; & \leq & \Pi -\rho N. \end{array}$$

Then, we have

$$\begin{array}{c} N\left(t\right)=\frac{\Pi }{\rho }+(N\left(0\right)-\frac{\Pi }{\rho })e^{-\rho t}. \end{array}$$ (8)

When *t* → ∞, we have $\frac{\Pi }{\rho }$. So, the state variables given in system (6) are non-negative for any *t* ≥ 0. Thus, any solution associated with the model (6) is continuously positive for any *t* ≥ 0. Therefore, the model (6) is properly defined and has epidemiological significance, and its dynamics can be studied in the region given below,

$$\begin{array}{c} \Omega =\{Z\in R_{5}^{+}:S+I+A+T+R\leq \frac{\Pi }{\rho }\}, \end{array}$$ (9)

in which *Z* = (*S*, *I*, *A*, *T*, *R*).

**Lemma 1**. *Given that the initial data of the model* (6) *at t* = 0, *is S*(0) > 0, *I*(0) > 0, *A*(0) > 0, *T*(0) > 0, *R*(0) > 0, *then for* ∀*t* > 0, *every solution of the model is non-negative*.

*Proof*. Let *t*_1_ = sup{*t* > 0: *S* > 0, *I* > 0, *A* > 0, *T* > 0, *R* > 0 ∈ [0, *t*]}. *t*_1_ > 0. It follows from the first equation of the system (6) that

$$\begin{array}{ccc} \frac{dS(t)}{dt} & = & \Pi -(\Psi \left(t\right)+k_{4})S\left(t\right), \end{array}$$ (10)

where *k*_4_ = (*ω* + *ρ*). Arranging the above equation, we write

$$\begin{array}{ccc} \frac{dS(t)}{dt}+\left(\Psi \left(t\right)+k_{4}\right)S\left(t\right) & = & \Pi . \end{array}$$

Utilizing the integration factor, we obtain

$$\begin{array}{c} \frac{d}{dt}[S\left(t\right)\text{exp}(k_{4}t+\int_{0}^{t}\Psi \left(z\right)dz)]=\Pi \text{exp}(k_{4}t+\int_{0}^{t}\Psi \left(z\right)dz). \end{array}$$

We arrive at the following results,

$$\begin{array}{c} \begin{array}{cc} S(t_{1})= & S\left(0\right)\text{exp}(-k_{4}t_{1}-\int_{0}^{t_{1}}\Psi \left(z\right)dz)\\ & +\text{exp}(-k_{4}t_{1}-\int_{0}^{t_{1}}\Psi \left(z\right)dz)\\ & \times \Pi \int_{0}^{t_{1}}\text{exp}(k_{4}x+\int_{0}^{x}\Psi \left(z\right)dz)dx>0. \end{array} \end{array}$$

We show *I* > 0, for this we write

$$\begin{array}{cc} \frac{dI(t)}{dt} & =\Psi S+\nu_{1}T-k_{1}I,\\ \; & \geq -k_{1}I. \end{array}$$

Further simplifications leads to $I(t)\geq I(0)e^{-k_{1}t}$. Similarly, *A* > 0, *T* > 0, *R* > 0 and we get $A(t)>A_{0}e^{-k_{2}t}$, $T(t)\geq T(0)e^{-k_{3}t}$, *R*(*t*) ≥ *R*(0)*e*^−*ρt*^. So, all the state variables given in the system (6), i.e, *S*(*t*) > 0, *I*(*t*) > 0, *A*(*t*) > 0, *T*(*t*) > 0, *R*(*t*) > 0.

## 3 Analysis of the equilibrium points

The analysis of the equilibrium points of the model (6) shall be considered in this section. We first, obtain the DFE, represented by $U_{0}$, and is shown by:

$$\begin{array}{c} U_{0}=(\frac{\Pi }{k_{4}},0,0,0,\frac{\omega \Pi }{\rho k_{4}}). \end{array}$$

We calculate the basic reproduction number (BRN) associated with the model (6), usually denoted by $R_{0}$. The next-generation matrix approach presented in the work given in [47] will be considered. To get the basic reproduction number, we consider the equations of the model (6) given by,

$$\begin{array}{c} \left\{ \begin{array}{c} \frac{dI}{dt}=\Psi S+\nu_{1}T-\left(\rho +\psi_{1}+\psi_{2}\right)I,\\ \frac{dA}{dt}=\psi_{1}I-\left(d_{1}+\rho \right)A+\nu_{2}T,\\ \frac{dT}{dt}=\psi_{2}I-\left(\rho +d_{2}+\nu_{1}+\nu_{2}\right)T. \end{array}\right. \end{array}$$ (11)

The system (11) is splitted into two parts accoridng to [47], and are given by

$$\begin{array}{c} F=(\begin{array}{c} \Psi S\\ 0\\ 0 \end{array}),\;and\;V=(\begin{array}{c} -\nu_{1}T+\left(\rho +\psi_{1}+\psi_{2}\right)I\\ -\psi_{1}I+\left(d_{1}+\rho \right)A+\nu_{2}T\\ -\psi_{2}I+\left(\rho +d_{2}+\nu_{1}+\nu_{2}\right)T. \end{array}). \end{array}$$

Further, we get the following,

$$\begin{array}{c} F=(\begin{array}{ccc} \frac{\tau S^{0}}{S^{0}+R^{0}} & \frac{\tau \beta_{2}S^{0}}{S^{0}+R^{0}} & \frac{\tau \beta_{1}S^{0}}{S^{0}+R^{0}}\\ 0 & 0 & 0\\ 0 & 0 & 0 \end{array}),\;\text{and}\;\;V=(\begin{array}{ccc} k_{1} & 0 & -\nu_{1}\\ -\psi_{1} & k_{2} & -\nu_{2}\\ -\psi_{2} & 0 & k_{3} \end{array}). \end{array}$$

Taking the inverse of *V* and then using the spectral radius of $\{\bar{\rho }\left(FV^{-1}\right)$, we obtain the $R_{0}=\{\bar{\rho }\left(FV^{-1}\right)$, and $\bar{\rho }$ indicates the spectral radius, the mathematical expression for $R_{0}$ is obtained as below:

$$\begin{array}{cc} R_{0} & =R_{1}+R_{2}+R_{3}+R_{4}+R_{5},\\ \; & =\frac{\beta_{2}\nu_{2}\rho \tau \psi_{2}}{k_{1}k_{2}k_{3}k_{4}}+\frac{\beta_{2}\rho \tau \psi_{1}}{k_{1}k_{2}k_{4}}+\frac{\beta_{1}\rho \tau \psi_{2}}{k_{1}k_{3}k_{4}}+\frac{\nu_{1}\psi_{2}}{k_{1}k_{3}}+\frac{\rho \tau }{k_{1}k_{4}}, \end{array}$$

where *k*_1_ = (*ρ* + *ψ*_1_ + *ψ*_2_), *k*_2_ = (*d*_1_ + *ρ*), *k*_3_ = (*d*_2_ + *ν*_1_ + *ν*_2_ + *ρ*), *k*_4_ = (*ρ* + *ω*).

We carry out the model’s local asymptotical stability around the DFE $U_{0}$ in the theorem presented below.

**Theorem 1**. *The DFE* $U_{0}$ *of* (6) *is LAS when* $R_{0}<1$.

*Proof*. At $U_{0}$, the Jacobian matrix for model (6) is given as follows:

$$\begin{array}{c} J\left(U_{0}\right)=(\begin{array}{ccccc} -k_{4} & -\frac{\rho \tau }{k_{4}} & -\frac{\rho \tau \beta_{2}}{k_{4}} & -\frac{\rho \tau \beta_{1}}{\rho +\omega } & 0\\ 0 & \frac{\rho \tau }{k_{4}}-k_{1} & \frac{\rho \tau \beta_{2}}{k_{4}} & \frac{\rho \tau \beta_{1}}{k_{4}}+\nu_{1} & 0\\ 0 & \psi_{1} & -k_{2} & \nu_{2} & 0\\ 0 & \psi_{2} & 0 & -k_{3} & 0\\ \omega & 0 & 0 & 0 & -\rho \end{array}). \end{array}$$

Three eigenvalues −*k*_4_, and −*ρ* in $J(U_{0})$ are negative, while the following equation can provide the rest,

$$\begin{array}{c} \lambda^{3}+u_{1}\lambda^{2}+u_{2}\lambda +u_{3}=0, \end{array}$$ (12)

where

$$\begin{array}{ccc} u_{1} & = & k_{1}(1-R_{5})+k_{2}+k_{3},\\ u_{2} & = & k_{1}k_{3}(1-R_{3}-R_{4}-R_{5})+k_{1}k_{2}(1-R_{2}-R_{5})+k_{2}k_{3},\\ u_{3} & = & k_{1}k_{2}k_{3}(1-R_{0}). \end{array}$$

Her, *u*_1_ > 0, due to the reason $R_{5}<R_{0}$ and *u*_2_ > 0 as $\left(R_{3}+R_{4}+R_{5}\right)<R_{0}<1$, and also $\left(R_{2}+R_{5}\right)<R_{0}<1$. We have *u*_3_ > 0, when $R_{0}<1$. All the coefficients *u*_1_, *u*_3_, and *u*_3_ are positive, further, it can be shown easily that *u*_1_*u*_2_ − *u*_3_ > 0. Then the Eq (12) provides eigenvalues with negative real parts and hence the system at $U_{0}$ is LAS if $R_{0}<1$.

**Theorem 2**. *When* $R_{0}\leq 1$, *model* (6) *at* $U_{0}$ *is GAS*.

*Proof*. Using the Lyapunov function defined below,

$$\begin{array}{c} K\left(t\right)=g_{1}(S-S^{0}-S^{0}\;\text{ln}(\frac{S}{S^{0}}))+g_{2}I+g_{3}A+g_{4}T. \end{array}$$ (13)

Taking the time derivative of K(t) and then considering the equations of the model (6), we get

$$\begin{array}{cc} K^{\prime }\left(t\right)= & g_{1}(1-\frac{S^{0}}{S})\left[\Pi -\tau \left(I+\beta_{1}T+\beta_{2}A\right)\frac{S}{N}-k_{4}S\right]\\ \; & +g_{2}\left[\tau \left(I+\beta_{1}T+\beta_{2}A\right)\frac{S}{N}+\nu_{1}T-k_{1}I\right]\\ \; & +g_{3}\left[\psi_{1}I-k_{2}A+\nu_{2}T\right]+g_{4}\left[\psi_{2}I-k_{3}T\right]. \end{array}$$

The following is obtained after some arrangement,

$$\begin{array}{cc} K^{\prime }\left(t\right)= & -g_{1}k_{4}\frac{{\left(S-S^{0}\right)}^{2}}{S}+\left(g_{2}-g_{1}\right)\left[\tau \left(I+\beta_{1}T+\beta_{2}A\right)\frac{S}{N}\right]\\ \; & +[\frac{g_{1}\tau S^{0}}{N^{0}}+g_{3}\psi_{1}+g_{4}\psi_{2}-g_{2}k_{1}]I\\ \; & +[\frac{g_{1}\tau \beta_{1}S^{0}}{N^{0}}+g_{2}\nu_{1}+g_{3}\nu_{2}-g_{4}k_{3}]T\\ \; & +[\frac{g_{1}\tau \beta_{2}S^{0}}{N^{0}}-g_{3}k_{2}]A. \end{array}$$

Now, letting the values of the constants, *g*_1_ = *g*_2_ = *k*_2_*k*_3_*k*_4_, *g*_3_ = *k*_3_*β*_2_*ρτ*, and *g*_4_ = *β*_2_*ν*_2_*ρτ* + *k*_2_*β*_1_*ρτ* + *ν*_1_*k*_2_*k*_4_, the following is obtained,

$$\begin{array}{ccc} K^{\prime }\left(t\right) & \leq & -k_{2}k_{3}k_{4}^{2}\frac{{\left(S-S^{0}\right)}^{2}}{S}+k_{1}k_{2}k_{3}k_{4}\left(R_{0}-1\right)I. \end{array}$$

Here, $K^{\prime }\left(t\right)=0$ when *S* = *S*^0^ and *I* = *T* = *A* = 0. When using *I* = *T* = *A* = 0 in equations of the model (6) for *t* → ∞, then we have the DFE $U_{0}$. Consequently, the DEF $U_{0}$ is GAS for $R_{0}\leq 1$ based on LaSalle’s invariance principle.

### 3.1 Existence of equilibrium

Here, we shall examine the HIV/AIDS model (6) to find out the existence of endemic equilibrium (EE). We describe the EE of the system (6) by $U_{1}=\left(S^{*},I^{*},A^{*},T^{*},R^{*}\right)$, where

$$\begin{array}{ccc} S^{*} & = & \frac{\Pi }{\Psi^{*}+k_{4}},\\ I^{*} & = & \frac{\nu_{1}T^{*}+\Psi^{*}S^{*}}{k_{1}},\\ A^{*} & = & \frac{\psi_{1}I^{*}+\nu_{2}T^{*}}{k_{2}},\\ T^{*} & = & \frac{\psi_{2}I^{*}}{k_{3}},\\ R^{*} & = & \frac{\omega S^{*}}{\rho }, \end{array}$$ (14)

where

$$\begin{array}{c} \Psi^{*}=\frac{\tau (I^{*}+\beta_{1}T^{*}+\beta_{2}A^{*})}{N^{*}}. \end{array}$$

Using the values from (14) into Ψ*, then, we obtain the following,

$$\begin{array}{c} c_{1}\lambda^{*}+c_{2}=0, \end{array}$$

where

$$\begin{array}{ccc} c_{1} & = & \rho \psi_{2}(k_{2}+\nu_{2})+k_{3}\rho (k_{2}+\psi_{1}),\\ c_{2} & = & k_{1}k_{2}k_{3}k_{4}\left(1-R_{0}\right). \end{array}$$ (15)

It follows from (15) that *c*_1_ > 0 and *c*_2_ is positive whenever $R_{0}<1$. Further, λ* = −*c*_2_/*c*_1_, and $R_{0}>1$, we get the positive existence of unique EE. The obtained equilibria imply that there is no existing phenomenon of backward bifurcation in the proposed HIV/AIDS system.

### 3.2 Global stability

We shall present below the GAS of the system (6) at $U_{1}$. The following are obtained at $U_{1}$ of the model at steady-state, which will be used in the proof:

$$\begin{array}{c} \left\{ \begin{array}{c} \Pi =\Psi S^{*}+k_{4}S^{*},\\ \Psi S^{*}+\nu_{1}T^{*}=k_{1}I^{*},\\ \psi_{1}I^{*}=k_{2}A^{*}+\nu_{2}T^{*},\\ \psi_{2}I^{*}=k_{3}T^{*}. \end{array}\right. \end{array}$$

**Theorem 3**. *When* $R_{0}>1$, *the EE of system* (6) *is GAS*.

*Proof.* The following defines the Lyapunov function:

$$\begin{array}{cc} L= & \left(S-S^{*}-S^{*}\;\text{log}(\frac{S}{S^{*}}))+(I-I^{*}-I^{*}\;\text{log}(\frac{I}{I^{*}})\right)\\ \; & +\frac{\nu_{1}T^{*}}{\psi_{1}I^{*}}(A-A^{*}-A^{*}\;\text{log}(\frac{A}{A^{*}}))+\frac{\nu_{1}T^{*}}{\psi_{2}I^{*}}(T-T^{*}-T^{*}\;\text{log}(\frac{T}{T^{*}})). \end{array}$$ (16)

Differentiating Eq 16 along the model (6), we get

$$\begin{array}{ccc} L^{\prime } & = & (1-\frac{S^{*}}{S})S^{\prime }+(1-\frac{I^{*}}{I})I^{\prime }+\frac{\nu_{1}T^{*}}{\psi_{1}I^{*}}(1-\frac{A^{*}}{A})A^{\prime }+\frac{\nu_{1}T^{*}}{\psi_{2}I^{*}}(1-\frac{T^{*}}{T})T^{\prime }. \end{array}$$ (17)

Each terms in (17) are calculated by

$$\begin{array}{ccc} (1-\frac{S^{*}}{S})S^{\prime } & = & (1-\frac{S^{*}}{S})\left[\Pi -\tau \left(I+\beta_{1}T+\beta_{2}A\right)S-k_{4}S\right],\\ \; & = & (1-\frac{S^{*}}{S})[\tau \left(I^{*}+\beta_{1}T^{*}+\beta_{2}A^{*}\right)S^{*}+k_{4}S^{*}\\ \; & \; & -\tau \left(I+\beta_{1}T+\beta_{2}A\right)S-k_{4}S],\\ \; & = & k_{4}S^{*}(2-\frac{S}{S^{*}}-\frac{S^{*}}{S})+(1-\frac{S^{*}}{S})[\tau \left(I^{*}+\beta_{1}T^{*}+\beta_{2}A^{*}\right)S^{*}\\ \; & \; & -\tau (I+\beta_{1}T+\beta_{2}A)S]\\ \; & = & k_{4}S^{*}(2-\frac{S}{S^{*}}-\frac{S^{*}}{S})+\tau \left(I^{*}+\beta_{1}T^{*}+\beta_{2}A^{*}\right)S^{*}\\ \; & \; & -\frac{S^{*}}{S}\tau \left(I^{*}+\beta_{1}T^{*}+\beta_{2}A^{*}\right)S^{*}\\ \; & \; & -\tau \left(I+\beta_{1}T+\beta_{2}A\right)S+\tau \left(I+\beta_{1}T+\beta_{2}A\right)S^{*}. \end{array}$$ (18)

$$\begin{array}{ccc} (1-\frac{I^{*}}{I})I^{\prime } & = & (1-\frac{I^{*}}{I})[\tau \left(I+\beta_{1}T+\beta_{2}A\right)S+\nu_{1}T-k_{2}I],\\ \; & = & (1-\frac{I^{*}}{I})[\tau \left(I+\beta_{1}T+\beta_{2}A\right)S+\nu_{1}T\\ \; & \; & -(\frac{\tau \left(I^{*}+\beta_{1}T^{*}+\beta_{2}A^{*}\right)S^{*}+\nu_{1}T^{*}}{I^{*}})I],\\ \; & = & \nu_{1}T^{*}(1-\frac{I}{I^{*}}+\frac{T}{T^{*}}-\frac{TI^{*}}{IT^{*}})+\tau \left(I+\beta_{1}T+\beta_{2}A\right)S\\ \; & \; & -\tau \left(I+\beta_{1}T+\beta_{2}A\right)\frac{S}{I}I^{*}+\tau \left(I^{*}+\beta_{1}T^{*}+\beta_{2}A^{*}\right)S^{*}\\ \; & \; & -\tau \left(I^{*}+\beta_{1}T^{*}+\beta_{2}A^{*}\right)S^{*}\frac{I}{I^{*}}. \end{array}$$ (19)

$$\begin{array}{ccc} \frac{\nu_{1}T^{*}}{\psi_{1}I^{*}}(1-\frac{A^{*}}{A})A^{\prime } & = & \frac{\nu_{1}T^{*}}{\psi_{1}I^{*}}(1-\frac{A^{*}}{A})\left[\psi_{1}I-k_{2}A+\nu_{2}T\right],\\ \; & = & \frac{\nu_{1}T^{*}}{\psi_{1}I^{*}}(1-\frac{A^{*}}{A})\left[\psi_{1}I+\nu_{2}T-\left(\frac{\psi_{1}I^{*}+\nu_{2}T^{*}}{A^{*}}\right)A\right],\\ \; & = & \nu_{1}T^{*}(1-\frac{A}{A^{*}}-\frac{A^{*}I}{AI^{*}}+\frac{I}{I^{*}})\\ \; & \; & +\frac{\nu_{1}T^{*}}{\psi_{1}I^{*}}\nu_{2}T^{*}(1+\frac{T}{T^{*}}-\frac{A}{A^{*}}-\frac{A^{*}T}{AT^{*}}). \end{array}$$ (20)

$$\begin{array}{ccc} \frac{\nu_{1}T^{*}}{\psi_{2}I^{*}}(1-\frac{T^{*}}{T})T^{\prime } & = & \frac{\nu_{1}T^{*}}{\psi_{2}I^{*}}(1-\frac{T^{*}}{T})\left[\psi_{2}I-k_{3}T\right],\\ \; & = & \frac{\nu_{1}T^{*}}{\psi_{2}I^{*}}(1-\frac{T^{*}}{T})\psi_{2}(I-\frac{I^{*}}{T^{*}}T),\\ \; & = & \nu_{1}T^{*}(1-\frac{T}{T^{*}}-\frac{IT^{*}}{TI^{*}}-\frac{I}{I^{*}}). \end{array}$$ (21)

Using the Eqs (18–21) into Eq (17), we have

$$\begin{array}{ccc} L^{\prime } & = & \left(k_{4}S^{*}+\tau I^{*}S^{*}\right)(2-\frac{S}{S^{*}}-\frac{S^{*}}{S})+\tau \left(\beta_{1}T^{*}+\beta_{2}A^{*}\right)S^{*}(2-\frac{S^{*}}{S}-\frac{I}{I^{*}})\\ \; & \; & +\tau \left(\beta_{1}T+\beta_{2}A\right)S^{*}(1-\frac{SI^{*}}{IS^{*}})\\ \; & \; & +\nu_{1}T^{*}(3-\frac{A}{A^{*}}-\frac{A^{*}I}{AI^{*}}-\frac{I}{I^{*}}-\frac{IT^{*}}{TI^{*}}-\frac{TI^{*}}{IT^{*}})\\ \; & \; & +\frac{\nu_{1}\nu_{2}{T^{*}}^{2}}{\psi_{1}I^{*}}(1-\frac{A}{A^{*}}-\frac{A^{*}T}{AT^{*}}+\frac{T}{T^{*}}) \end{array}$$ (22)

The terms inside the bracket ensure that *L*′[*t*] ≤ 0, and hence the model at EE is GAS.

## 4 Parameters estimations

Here, we analyze the actual data obtained from the cited website [48] on the reported incidences of HIV/AIDS cases in Pakistan between the years 1992 and 2020. To determine the numerical values of the fitted parameters, the proposed model (6) was fitted using the nonlinear least squares curve fitting method along with actual data. Various methods are available in the literature to analyze the data versus model fit, among these are least squares, Gaussian, and much more. In a recent study [49], the authors utilized the concept of data fitting to the COVID-19 model and obtained the estimated parameters with 95% CI. The data considered here is in years, so the time unit shall be considered per year. Since 1992 is the year that the study began considering the HIV/AIDS epidemic, 122.4 million people were thought to be living in Pakistan as of 1992; see for more details [50]. The average life span in Pakistan in 1992 was 1/60.12 per year. Based on the 1992 year, the initial population is *N*(0) = 122, 400, 000, as a result, the initial values for the remaining variables in the system (6) can be computed as follows: If there is no disease, then we can use *S*(0) = 122397990, here, *I*(0) = 10 is taken from data, which are the reported cases of HIV-infected people in 1992, *A*(0) = *T*(0) = *R*(0) = 0 as there is no AIDS, treated and recovered cases as on the disease starting. The data collection and analysis technique adhered to the terms and conditions set by the data provider.

**Fig 2:**
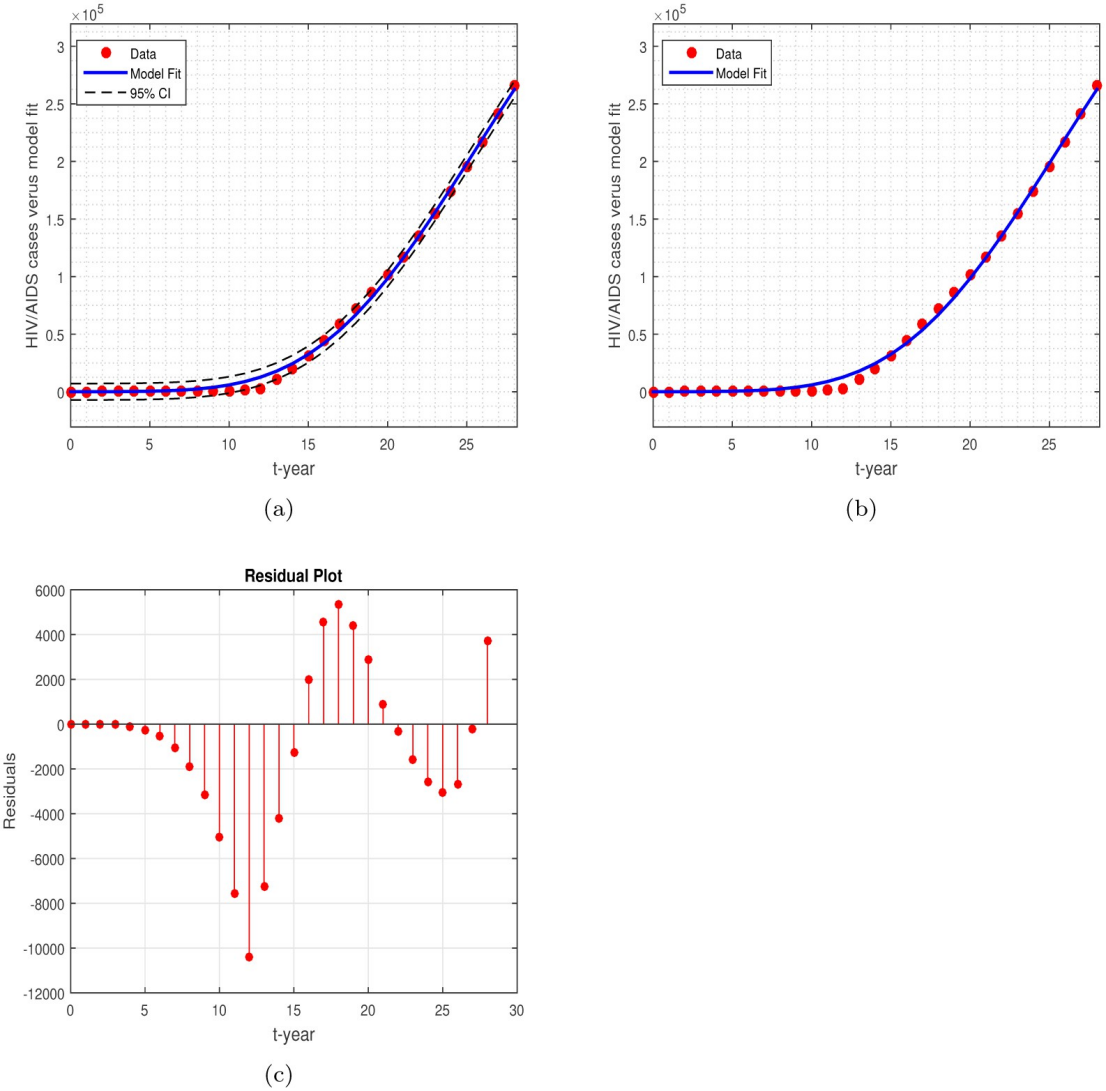
The graph shows the data fitting of HIV cases versus the model solution, the bold ‘dot’ defines the HIV cases, while the line shows the model solution: (a) model versus data fitting with 95%, (b) model versus data fit, and (c) the residual plot.

Among the number of model parameters, we calculate the birth rate directly from the equation Π = *ρ* × *N*(0), which is approximate Π = 2035928.14 per year per person generation in Pakistan in 1992, where *ρ* = 1/60.12 is per year life span in Pakistan at 1992. The rest of the right parameters were fitted to the model during the experiment and obtained their numerical values when getting a reasonable fit to the model (6). We used the ode45 algorithm, a built-in system in MATLAB, for data fitting. The fitted parameter was at a 95% confidence interval (CI). We have the BRN for the HIV/AIDS model based on the fitted parameters, with a 95% CI is $R_{0}\approx 0.8284$. Fig 2 shows the resulting data versus model output, which has a good correlation to the data. Fig 2b shows the fitting of the data to the model, while the 95% CI is shown in Fig 2a. Fig 2(c) is the corresponding residual plot. The goodness of fit is 0.9980. Table 3 shows the resulting output of the parameter values that were determined from the experiment.

**Table 3:** Parameters values.

Notation	Numeric value	95% CI	Ref.
Π	*ρ* × *N*(0)	–	Estimated
*ρ*	$\frac{1}{60.12}$	–	[51]
*τ*	1.2312	[1.163023, 1.462948]	Fitted
*ω*	0.0619	[0.035421, 0.092197]	Fitted
*ν*_1_	0.0929	[0.010204, 0.258353]	Fitted
*ψ*_1_	0.2456	[0.136914, 0.466263]	Fitted
*ψ*_2_	0.1330	[0.030554, 0.397416]	Fitted
*ν*_2_	0.4273	[0.068357, 0.650830]	Fitted
*d*_1_	0.2508	[0.010000, 0.819700]	Fitted
*d*_2_	0.3106	[0.097111, 0.629503]	Fitted
*β*_1_	0.3168	[0.182015, 0.464183]	Fitted
*β*_2_	0.2762	[0.196775, 0.356019]	Fitted

### 4.1 Sensitivity analysis

It is used to determine the sensitive parameters that either raise or lower the fundamental reproduction number. Finding such parameters that significantly affect the model is essential for disease curtailment. The realistic parameters obtained from the data fitting given in Table 3 will be considered to obtain the sensitivity analysis of $R_{0}$. For a general parameter *b* of $R_{0}$, the following sensitivity analysis formula is used to get sensitivity indices:

$$\begin{array}{c} \prod_{b}^{R_{0}}≔\frac{\partial R_{0}}{\partial b}\times \frac{b}{R_{0}}, \end{array}$$ (23)

Using the formula (23), the sensitive parameters to $R_{0}$ are shown in Table 4.

**Table 4:** Sensitivity analysis of $R_{0}$.

Parameter	*ρ*	*τ*	*β*_1_	*β*_2_
Sensitivity Index	0.698803	0.960861	0.0348047	0.226042
Parameter	*ν*_1_	*ν*_2_	*d*_1_	*d*_2_
Sensitivity Index	0.0257182	-0.0132479	-0.211983	-0.0448719
Parameter	*ψ*_1_	*ψ*_2_	*ω*	
Sensitivity Index	-0.443846	-0.214083	-0.75735	

**Fig 3:**
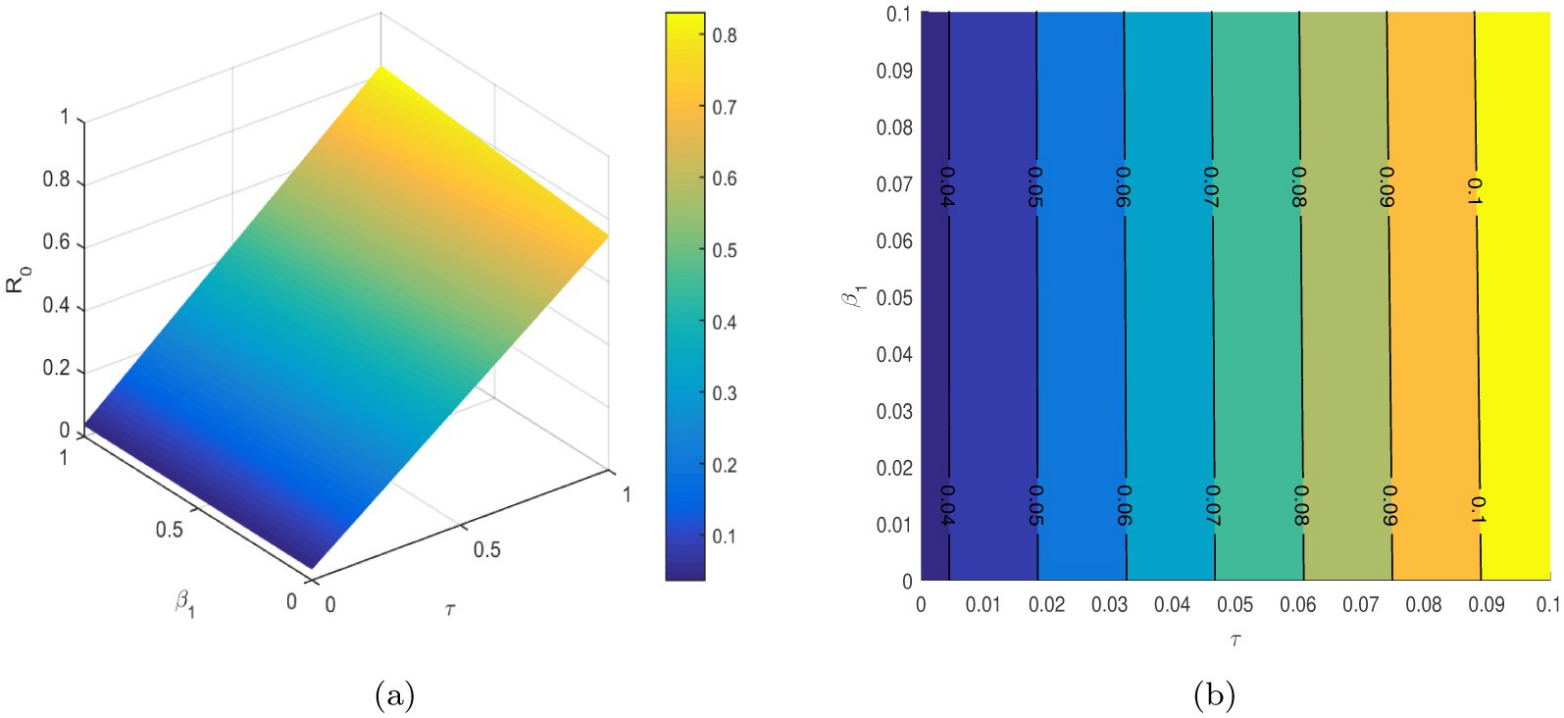
$R_{0}$ is a function of *τ* and *β*_1_, (a) 3D plot, and (b) a contour plot.

**Fig 4:**
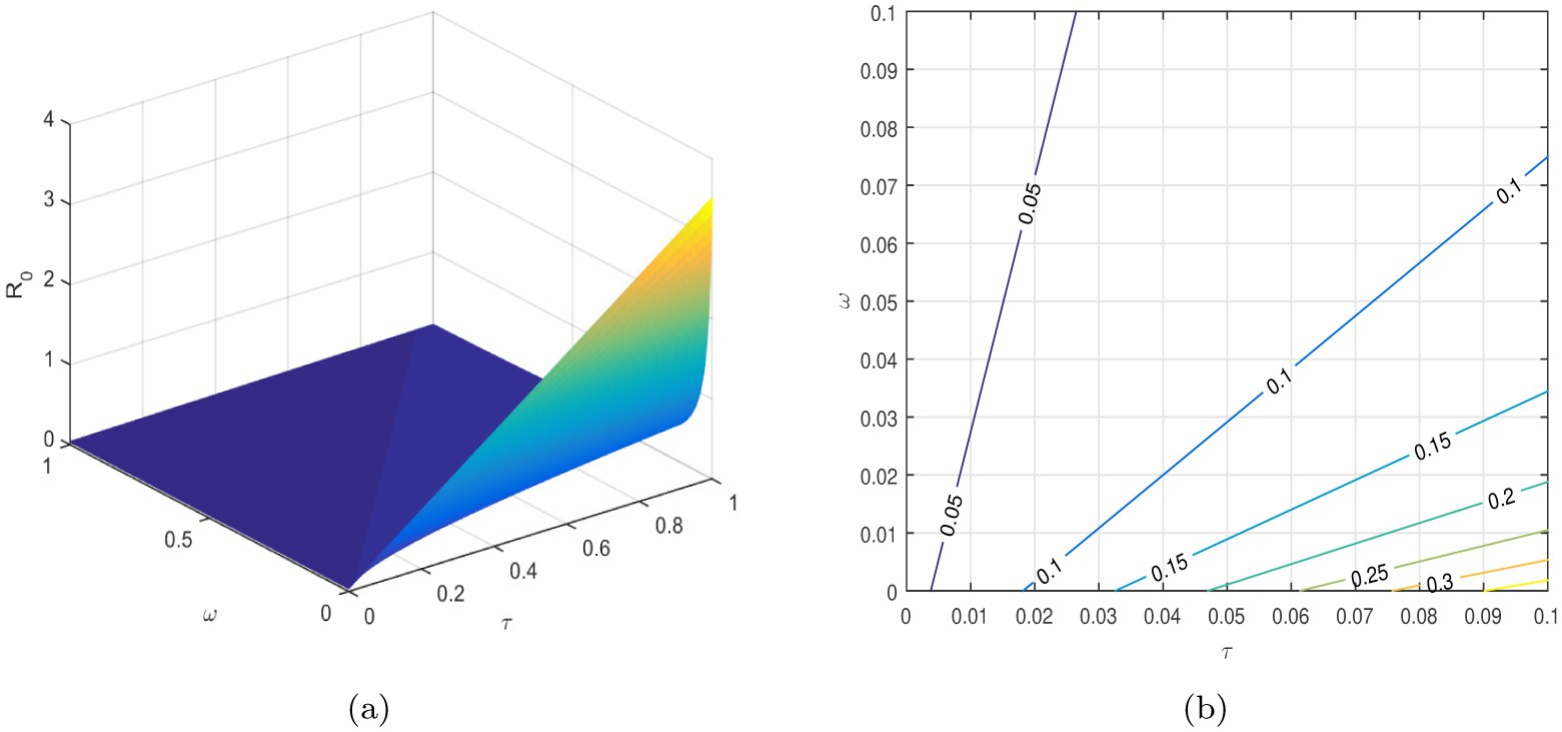
$R_{0}$ is a function of *τ* and *ω*, (a) 3D plot, and (b) a contour plot.

**Fig 5:**
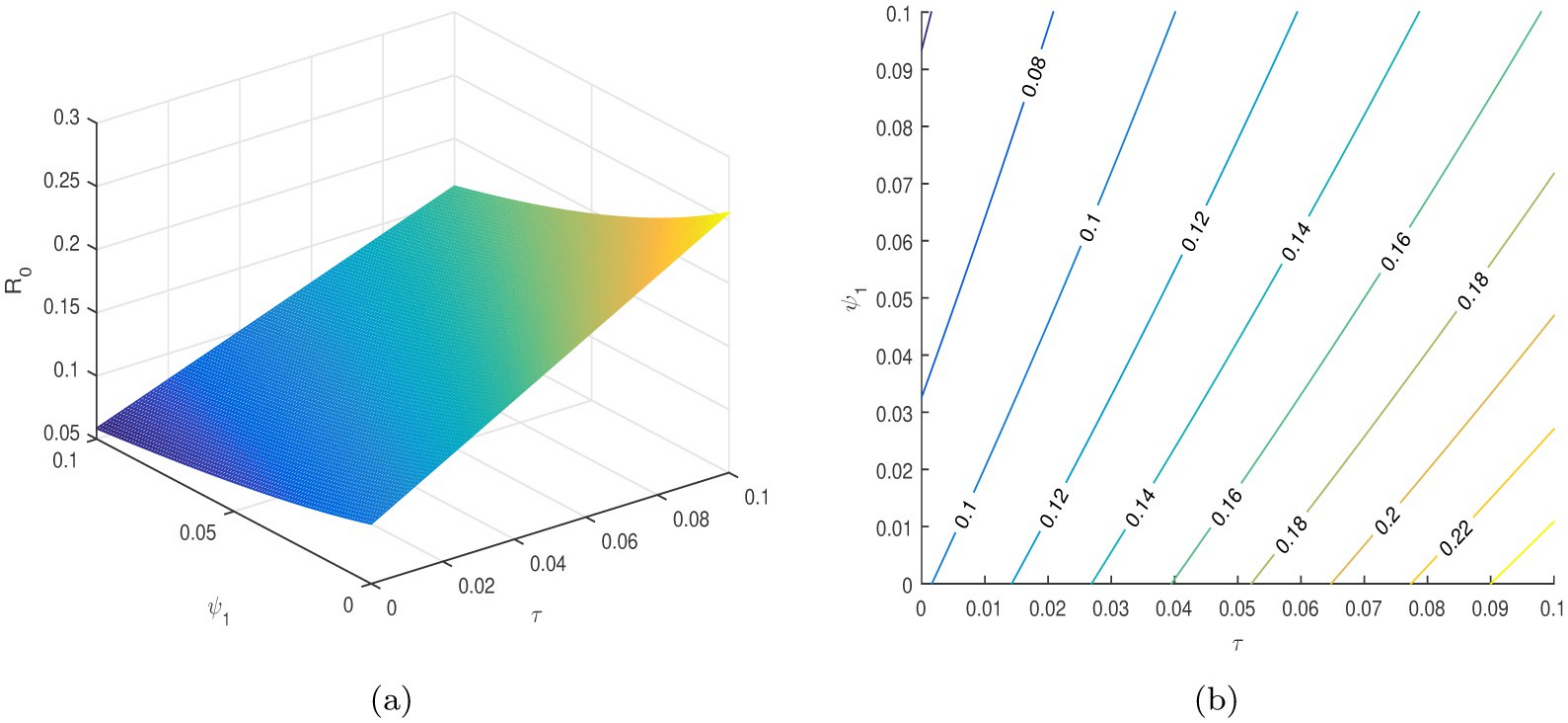
$R_{0}$ is a function of *τ*, and *ψ*_1_, (a) 3D plot, and (b) a contour plot.

**Fig 6:**
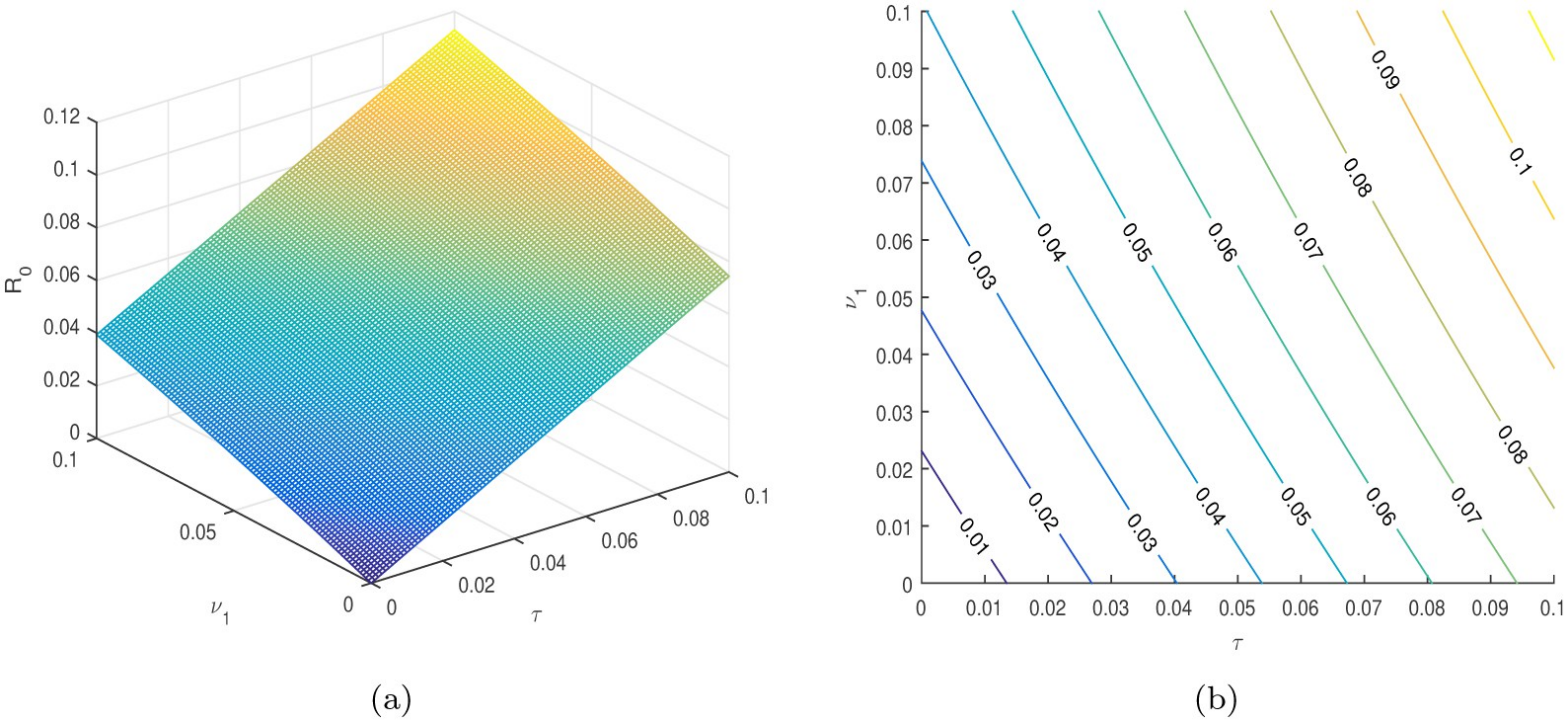
$R_{0}$ is a function of *τ* and *ν*_1_, (a) 3D plot, and (b) a contour plot.

**Fig 7:**
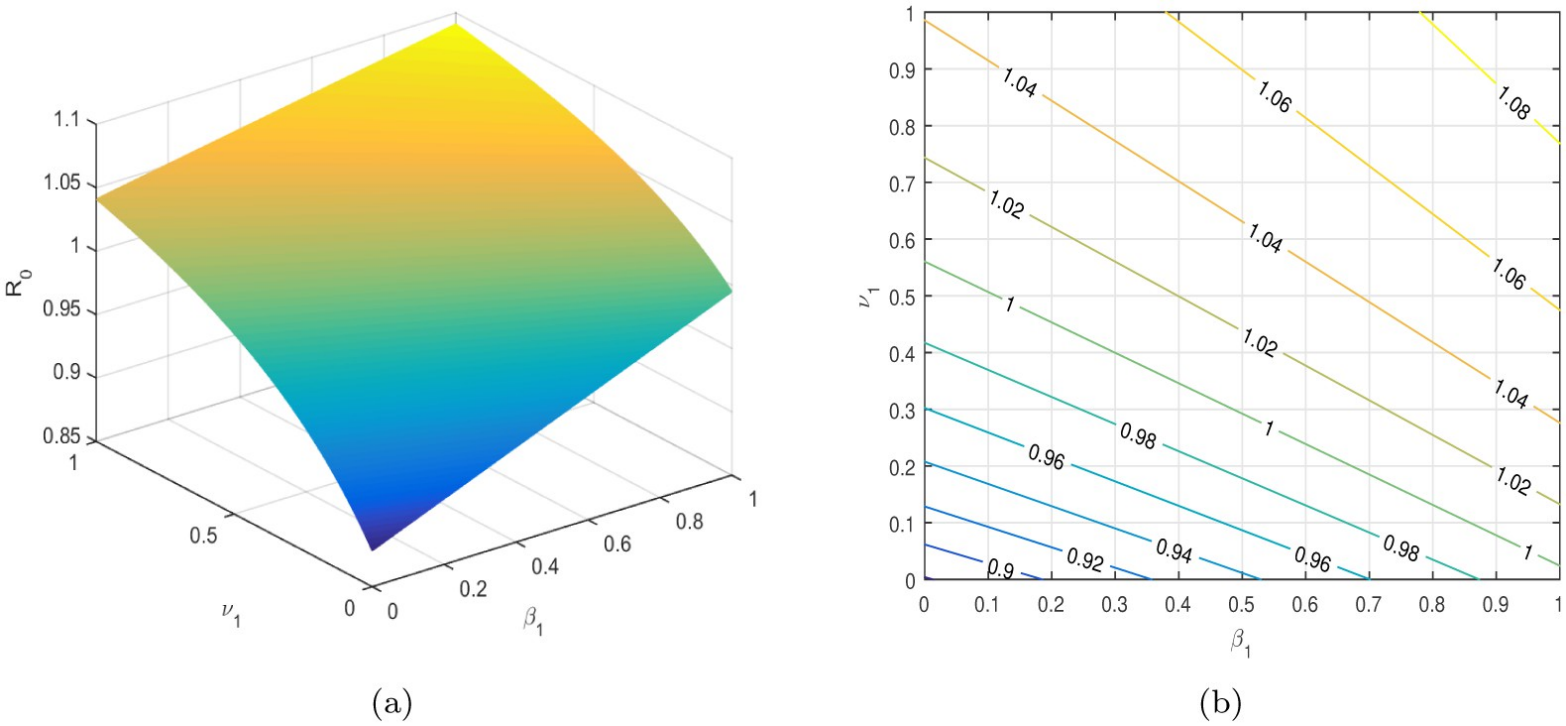
$R_{0}$ is a function of *β*_1_ and *ν*_1_, (a) 3D plot, and (b) a contour plot.

In Table 4, the parameters *τ*, *ω*, *ρ*, *ψ*_1_, *ψ*_2_, etc. are considered to be the most sensitive parameters that contribute to $R_{0}$. In the following graphical results shown in Figs 3–7, we consider the sensitive parameters as a function of $R_{0}$.

## 5 Numerical solution

**Fig 8:**
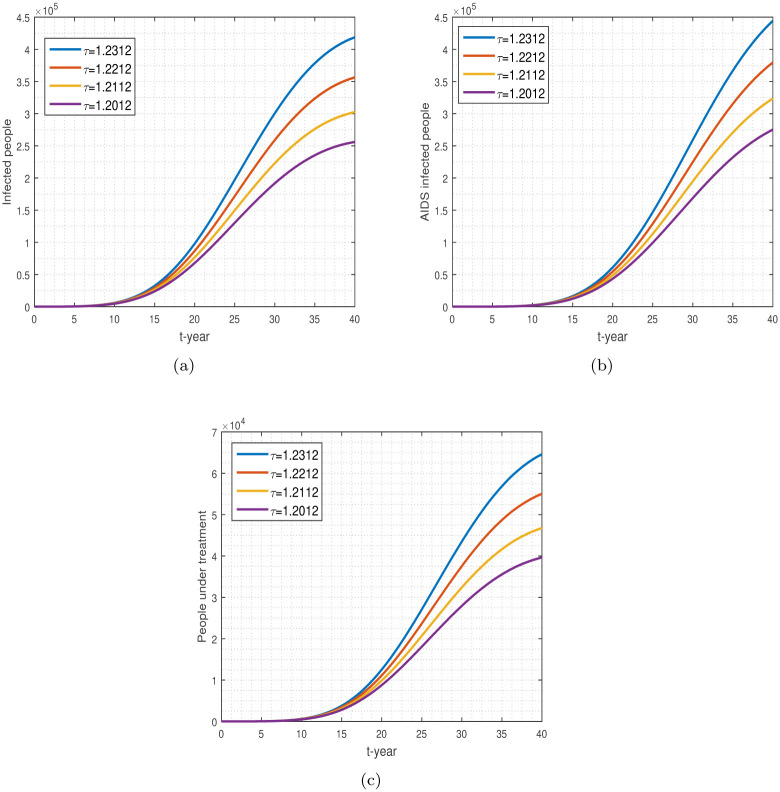
The plot shows the variation in *τ*. Numerical results for infected, AIDS-infected, and people under treatment are shown, respectively, by (a-c).

**Fig 9:**
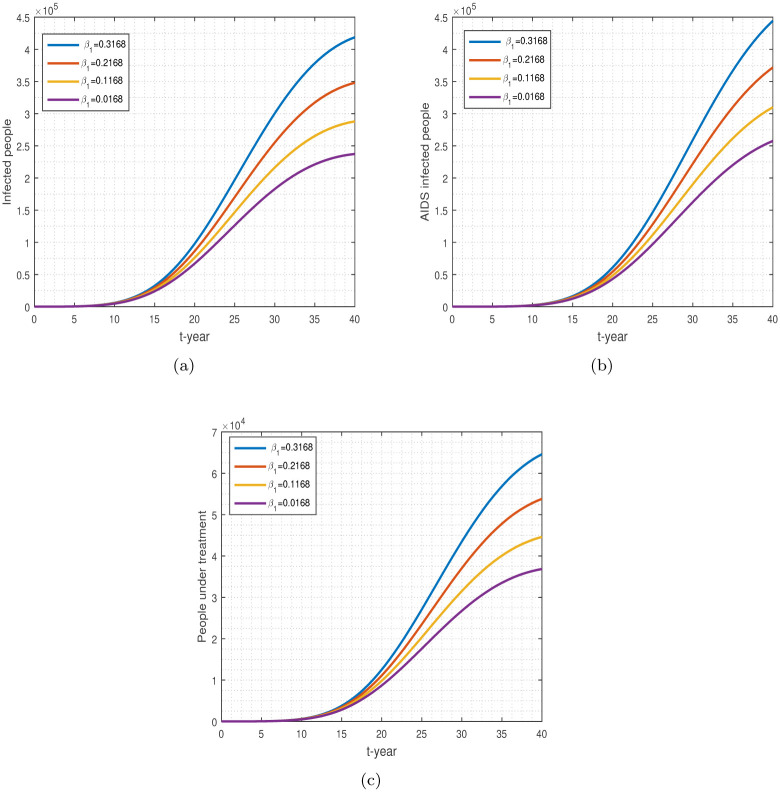
The impact of *β*_1_ on the components of the model. (a-c) shows respectively the infected, AIDS-infected, and treatment populations.

**Fig 10:**
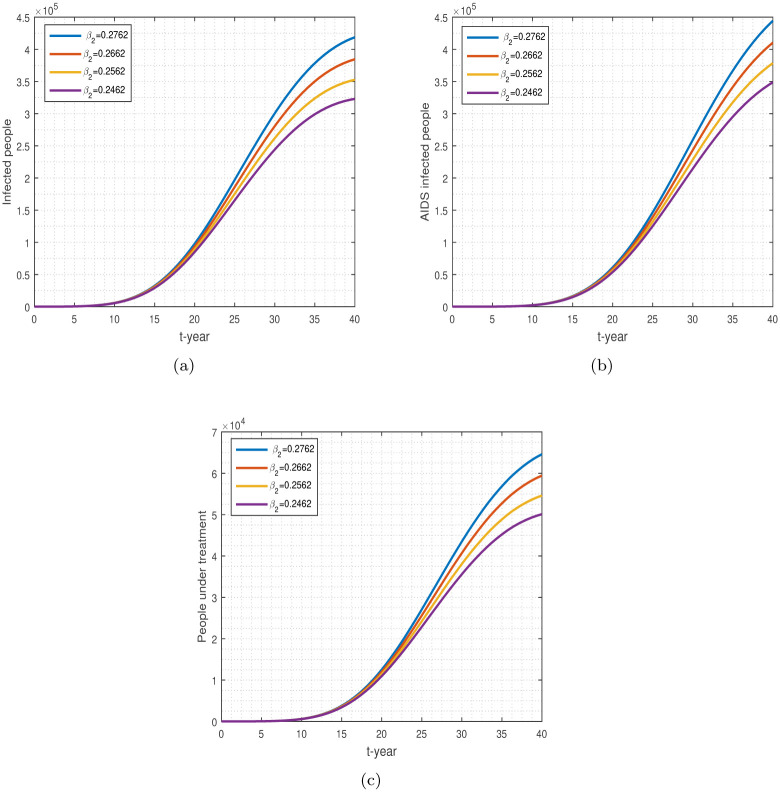
The impact of *β*_2_ on the disease compartment model: Sub-figures (a-c) denote, HIV, AIDS, and treatment populations.

**Fig 11:**
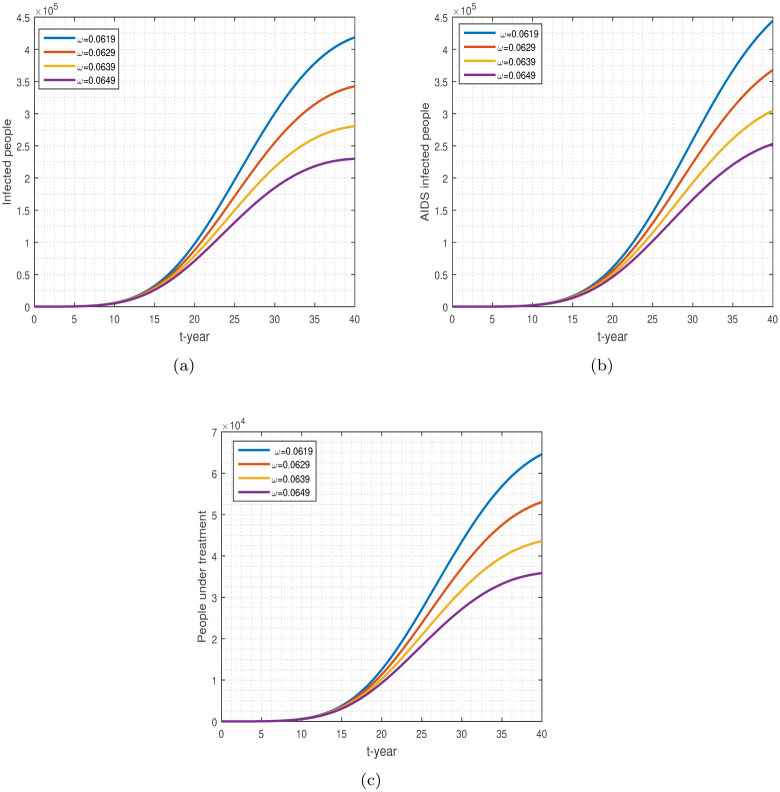
Individuals that follow safe sexual practices throughout their lives: Sub-figures (a-c) denote, HIV, AIDS, and treatment populations.

**Fig 12:**
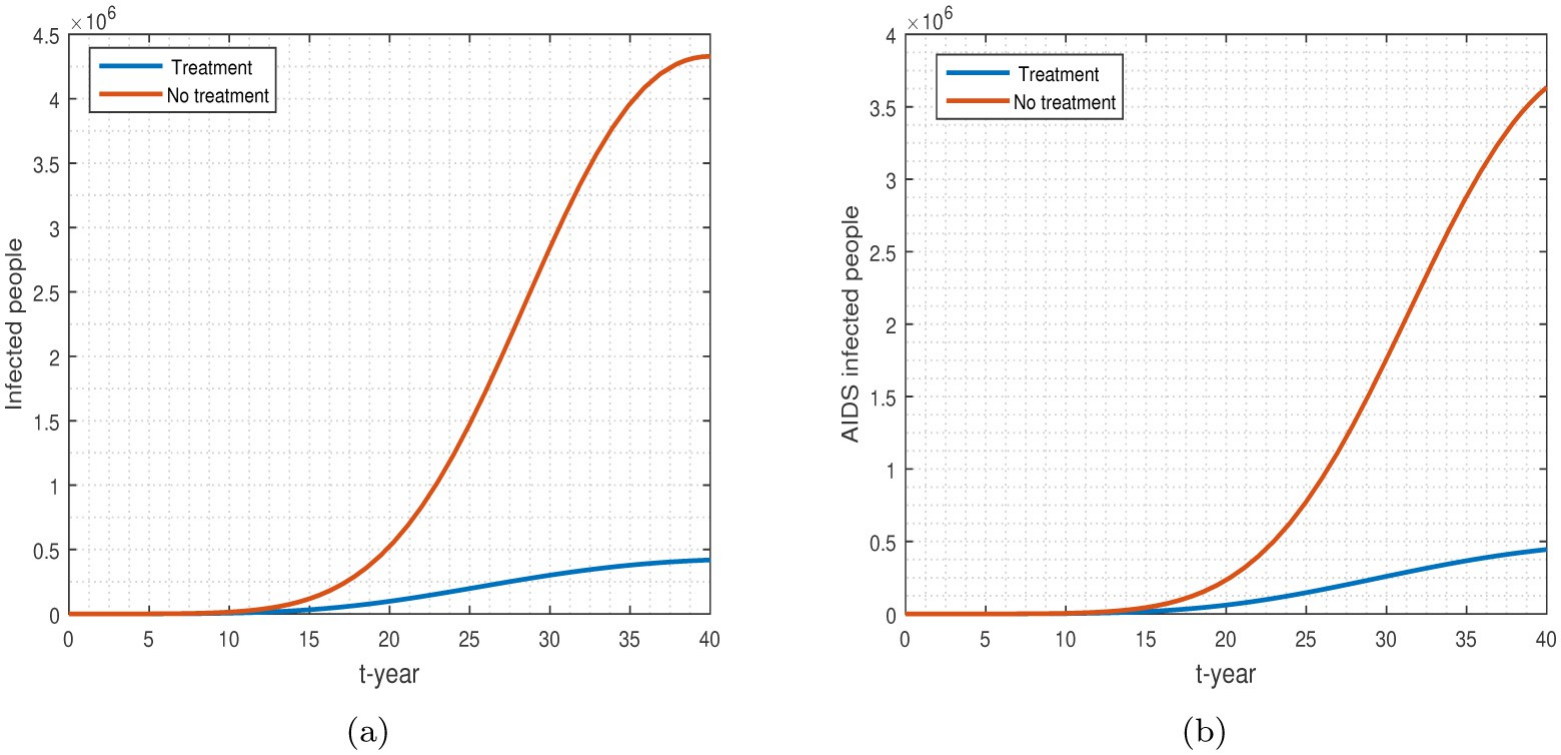
The model simulation of HIV infected and AIDS infected populations with *ψ*_2_ = 0.133 and without treatment *ψ*_2_ = 0. Subfigures: (a) HIV-infected, (b) AIDS-infected population.

We demonstrate the numerical simulation of the model (6) using the RK-4 (Runge-Kutta) scheme. The results are graphically displayed using the parameter values obtained from the data fitting technique illustrated in Table 3. Graphical results for the model solution based on the variation of parameters are shown in Figs 8–12.

Fig 8 shows the contact parameter *τ* and its effect on HIV-infected, AIDS-positive, and treatment-patient populations. Interactions between HIV-positive and healthy individuals can raise the population’s number of new cases. HIV may only be transmitted or spread by specific behaviors, such as injectable drug use or unprotected intercourse. Certain body fluids from an HIV-positive person are the only ones that can spread the virus, including breast milk, semen, rectal fluids, vaginal fluids, and pre-seminal fluids. Reducing the contact among healthy and HIV-infected people follows the aforementioned ways, we can control a better decrease in future cases, see the impact of *τ*, in Fig 8(a)–8(c).

Fig 9 represents the correlation between the parameter *β*_1_ and the number of individuals who are afflicted with HIV/AIDS and are undergoing treatment. By decreasing the parameter *β*_1_, the population of HIV infected, AIDS infected, and those under treatment is decreasing well. Individuals who utilize ART treatment can significantly decrease the disease burden in the community. ART treatment is beneficial for HIV patients to live longer, healthier lives. It also reduces the risk of HIV transmission and minimizes the chances of getting an AIDS infection.

The impact of parameter *β*_2_ on the population of infected, AIDS, and those under treatment is shown in Fig 10. It has been shown that decreasing the value of parameter *β*_2_ leads to a decrease in the number of individuals infected with HIV, affected by AIDS, and undergoing treatment. Reducing the interaction of healthy people with those of the infected, AIDS-infected, and those with treatment reduces disease transmission in the community.

The effect of parameter *ω* on the population with HIV/AIDS and those undergoing treatment is shown in Fig 11. Following safe sexual practices throughout their lives, the number of new cases in the community will decrease. Our result in Fig 11 indicates that by increasing the parameter *ω* (which means increasing sexually safe habits), the future cases generated by HIV, AIDS, and under-treatment cases will be significantly minimized.

The effects of the treatment on individuals infected with HIV and AIDS are shown in Fig 12. Fig 12 is displayed when comparing the model with treatment to the model without treatment. Individuals diagnosed with HIV will receive prompt treatment to limit the spread of the infection. While there is currently no cure for this infection, HIV management can effectively mitigate its impact. Usually, people are able to gain control over the illness within a span of six months.

## 6. Conclusion

An analysis of the dynamics of HIV/AIDS was conducted using data from actual reported cases. We conducted a thorough analysis of the model and found its relevant mathematical outcomes. The local and global asymptotical stability at the equilibrium points of the model are obtained and discussed. The equilibrium point $D_{0}$ is found locally stable when $R_{0}<1$. Further, a Lyapunov function is constructed, and the global asymptotical stability of the model is shown at $D_{0}$ when $R_{0}\leq 1$. The endemic equilibria are obtained and analyzed. We found that the model possesses a unique $D_{*}$. The EE $D_{*}$ is found globally asymptotically stable for $R_{0}>1$. A nonlinear least squares fitting method that minimizes the infected cases and provides reasonable results for the model. The level of significance during the experiment of fitting was set to be 95% confidence interval (CI). The parameter values and their respective 95% CI were obtained and shown in Table 3. We calculated the numeric values of the BRN for the predicted parameters as $R_{0}\approx 0.8284$.

The RK-4 method is used to solve the model numerically, and the corresponding results are graphically presented. Numerous parameters are displayed, and their effects on the model solution are described. The results indicate, that HIV/AIDS cases are growing continuously, and it is required by the government to minimize the contact between infected people with HIV and healthy people. Every individual should be educated about HIV/AIDS, its prevention, and early treatment to avoid the infection. The results indicate that by implementing the World Health Organization’s (WHO) recommended preventive measures, HIV/AIDS cases can be decreased and prevented. Our model could be helpful to policymakers in creating better strategies for managing the epidemic. The model determines the factors influencing disease transmission that will also help health care services, nongovernmental organizations, and other concerned organizations in the fight against HIV/AIDS.

## Supporting information

S1 Data (XLSX)

## References

[1] Palella FJ Jr, Delaney KM, Moorman AC, Loveless MO, Fuhrer J, Satten GA, et al. Declining morbidity and mortality among patients with advanced human immunodeficiency virus infection. New England Journal of Medicine. 1998;338(13):853–860. doi: 10.1056/NEJM199803263381301 9516219

[2] Alamer E, Zhong C, Hajnik R, Soong L, Hu H. Modulation of BRD4 in HIV epigenetic regulation: Implications for finding an HIV cure. Retrovirology. 2021;18(1):1–9. doi: 10.1186/s12977-020-00547-9 33413475 PMC7792063

[3] Kandwal R, Garg PK, Garg RD. Health GIS and HIV/AIDS studies: Perspective and retrospective. Journal of biomedical informatics. 2009;42(4):748–755. doi: 10.1016/j.jbi.2009.04.008 19426832

[4] Naresh R, Sharma D, et al. An HIV/AIDS model with vertical transmission and time delay. World J Model Simul. 2011;7(3):230–240.

[5] Walensky RP, Paltiel AD, Losina E, Mercincavage LM, Schackman BR, Sax PE, et al. The survival benefits of AIDS treatment in the United States. The Journal of infectious diseases. 2006;194(1):11–19. doi: 10.1086/505147 16741877

[6] Lou J, Lou Y, Wu J. Threshold virus dynamics with impulsive antiretroviral drug effects. Journal of mathematical biology. 2012;65:623–652. doi: 10.1007/s00285-011-0474-9 21987085 PMC3887123

[7] Chibaya S, Kgosimore M, Massawe ES. Mathematical analysis of drug resistance in vertical transmission of HIV/AIDS. Open Journal of Epidemiology. 2013;2013.

[8] Al-Fifi Z, Saleh NA, Elhaes H, Ibrahim M. On the molecular modeling analyses of novel HIV-1 protease inhibitors based on modified chitosan dimer. International Journal of Spectroscopy. 2015;2015. doi: 10.1155/2015/174098

[9] Naresh R, Tripathi A, Omar S. Modelling the spread of AIDS epidemic with vertical transmission. Applied Mathematics and Computation. 2006;178(2):262–272. doi: 10.1016/j.amc.2005.11.041

[10] Waziri AS, Massawe ES, Makinde OD. Mathematical modelling of HIV/AIDS dynamics with treatment and vertical transmission. Appl Math. 2012;2(3):77–89.

[11] Islam R, Ahmad R, Ghailan K, Hoque KE. An Islamic microfinance approach to scaling up the economic life of vulnerable people with HIV/AIDS in the Muslim society. Journal of religion and health. 2020;59:1327–1343. doi: 10.1007/s10943-019-00832-8 31134517

[12] Kumar S, Tadakamadla J, Areeshi AYBH, Tobaigy HAWM. Knowledge and attitudes towards HIV/AIDS among dental students of Jazan University, Kingdom Saudi Arabia. The Saudi dental journal. 2018;30(1):47–52. doi: 10.1016/j.sdentj.2017.10.003 30166871 PMC6112364

[13] Gosadi IM. National screening programs in Saudi Arabia: overview, outcomes, and effectiveness. Journal of infection and public health. 2019;12(5):608–614. doi: 10.1016/j.jiph.2019.06.001 31248815

[14] Darraj MA, Abdulhaq AA, Yassin A, Mubarki S, Shalaby HM, Keynan Y, et al. Tuberculosis among people living with HIV/AIDS in Jazan Region, Southwestern Saudi Arabia. Journal of infection and public health. 2021;14(11):1571–1577. doi: 10.1016/j.jiph.2021.09.009 34656963

[15] Ilyas M, Asad S, Ali L, Shah M, Badar S, Sarwar MT, et al. A situational analysis of HIV and AIDS in Pakistan. Virology journal. 2011;8:1–3. doi: 10.1186/1743-422X-8-191 21518454 PMC3107810

[16] Asia A. Pakistan sitting on a ticking AIDS bomb. Pukaar the journal of Naz Foundation International. 2007;56:16–17.

[17] Yusuf TT, Benyah F. Optimal strategy for controlling the spread of HIV/AIDS disease: a case study of South Africa. Journal of biological dynamics. 2012;6(2):475–494. doi: 10.1080/17513758.2011.628700 22873601

[18] Huo HF, Feng LX. Global stability for an HIV/AIDS epidemic model with different latent stages and treatment. Applied Mathematical Modelling. 2013;37(3):1480–1489. doi: 10.1016/j.apm.2012.04.013

[19] Huo HF, Chen R, Wang XY. Modelling and stability of HIV/AIDS epidemic model with treatment. Applied Mathematical Modelling. 2016;40(13-14):6550–6559. doi: 10.1016/j.apm.2016.01.054

[20] Liu H, Zhang JF. Dynamics of two time delays differential equation model to HIV latent infection. Physica A: Statistical Mechanics and its Applications. 2019;514:384–395. doi: 10.1016/j.physa.2018.09.087

[21] Dutta A, Gupta PK. A mathematical model for transmission dynamics of HIV/AIDS with effect of weak CD4+ T cells. Chinese journal of physics. 2018;56(3):1045–1056. doi: 10.1016/j.cjph.2018.04.004

[22] Li YM, Ullah S, Khan MA, Alshahrani MY, Muhammad T. Modeling and analysis of the dynamics of HIV/AIDS with non-singular fractional and fractal-fractional operators. Physica Scripta. 2021;96(11):114008. doi: 10.1088/1402-4896/ac15c3

[23] Gupta PK, Dutta A. A mathematical model on HIV/AIDS with fusion effect: Analysis and homotopy solution. The European Physical Journal Plus. 2019;134(6):265. doi: 10.1140/epjp/i2019-12599-8

[24] Jia J, Qin G. Stability analysis of HIV/AIDS epidemic model with nonlinear incidence and treatment. Advances in Difference Equations. 2017;2017:1–13. doi: 10.1186/s13662-017-1175-5

[25] Khan MA, Odinsyah HP, et al. Fractional model of HIV transmission with awareness effect. Chaos, Solitons & Fractals. 2020;138:109967. doi: 10.1016/j.chaos.2020.109967

[26] Ayele TK, Goufo EFD, Mugisha S. Mathematical modeling of HIV/AIDS with optimal control: a case study in Ethiopia. Results in Physics. 2021;26:104263. doi: 10.1016/j.rinp.2021.104263

[27] Espitia CC, Botina MA, Solarte MA, Hernandez I, Riascos RA, Meyer JF. Mathematical Model of HIV/AIDS Considering Sexual Preferences Under Antiretroviral Therapy, a Case Study in San Juan de Pasto, Colombia. Journal of Computational Biology. 2022;29(5):483–493. doi: 10.1089/cmb.2021.0323 35544039 PMC9125573

[28] Omondi E, Mbogo R, Luboobi L. A mathematical model of HIV transmission between commercial sex workers and injection drug users. Research in Mathematics. 2022;9(1):2082044. doi: 10.1080/27684830.2022.2082044

[29] Gao M, Jiang D, Hayat T. Qualitative Analysis of an HIV/AIDS Model with Treatment and Nonlinear Perturbation. Qualitative theory of dynamical systems. 2022;21(3):85. doi: 10.1007/s12346-022-00615-9

[30] Gao M, Jiang D, Hayat T. HIV/AIDS-pneumonia codynamics model analysis with vaccination and treatment Computational and Mathematical Methods in Medicine. 2022;2022:ID 3105734. 10.1155/2022/3105734 PMC8767370 35069778

[31] Teklu SW. Investigating the Effects of Intervention Strategies on Pneumonia and HIV/AIDS Coinfection Model BioMed Research International. 2023;2023:ID 5778209. 10.1155/2023/5778209 PMC10703535 38075304

[32] Teklu SW. Analysis of HBV and COVID-19 coinfection model with intervention strategies Computational and Mathematical Methods in Medicine. 2023;2023. doi: 10.1155/2023/6908757 37811291 PMC10558273

[33] Teklu SW, Terefe BB, Mamo DK, Abebaw YF. Optimal control strategies on HIV/AIDS and pneumonia co-infection with mathematical modelling approach Journal of Biological Dynamics. 2024;18(1):2288873. doi: 10.1080/17513758.2023.2288873 38140717

[34] Teklu SW. Impacts of optimal control strategies on the HBV and COVID-19 co-epidemic spreading dynamics Scientific Reports. 2024;14(1):5328. doi: 10.1038/s41598-024-55111-8 38438440 PMC10912759

[35] HIV/AIDS in Pakistan;.

[36] Akbar MA, Akinyemi L, Yao SW, Jhangeer A, Rezazadeh H, Khater MM, et al. Soliton solutions to the Boussinesq equation through sine-Gordon method and Kudryashov method. Results in Physics. 2021;25:104228. doi: 10.1016/j.rinp.2021.104228

[37] Akinyemi L, Rezazadeh H, Yao SW, Akbar MA, Khater MM, Jhangeer A, et al. Nonlinear dispersion in parabolic law medium and its optical solitons. Results in Physics. 2021;26:104411. doi: 10.1016/j.rinp.2021.104411

[38] Ahmad H, Akgül A, Khan TA, Stanimirović PS, Chu YM. New perspective on the conventional solutions of the nonlinear time-fractional partial differential equations. Complexity. 2020;2020:1–10. doi: 10.1155/2020/8841718

[39] Ahmad H, Khan TA. Variational iteration algorithm-I with an auxiliary parameter for wave-like vibration equations. Journal of Low Frequency Noise, Vibration and Active Control. 2019;38(3-4):1113–1124. doi: 10.1177/1461348418823126

[40] Ahmad H, Khan TA, Stanimirović PS, Chu YM, Ahmad I. Modified variational iteration algorithm-II: convergence and applications to diffusion models. Complexity. 2020;2020:1–14. doi: 10.1155/2020/9285686

[41] Ahmad H, Khan TA, Cesarano C. Numerical solutions of coupled Burgers’ equations. Axioms. 2019;8(4):119. doi: 10.3390/axioms8040119

[42] Ahmad H, Seadawy AR, Khan TA. Study on numerical solution of dispersive water wave phenomena by using a reliable modification of variational iteration algorithm. Mathematics and Computers in Simulation. 2020;177:13–23. doi: 10.1016/j.matcom.2020.04.005

[43] Ahmad I, Ahmad H, Inc M, Yao SW, Almohsen B. Application of local meshless method for the solution of two term time fractional-order multi-dimensional PDE arising in heat and mass transfer. Thermal Science. 2020;24(Suppl. 1):95–105. doi: 10.2298/TSCI20S1095A

[44] Inc M, Khan MN, Ahmad I, Yao SW, Ahmad H, Thounthong P. Analysing time-fractional exotic options via efficient local meshless method. Results in Physics. 2020;19:103385. doi: 10.1016/j.rinp.2020.103385

[45] Li JF, Ahmad I, Ahmad H, Shah D, Chu YM, Thounthong P, Ayaz M. Numerical solution of two-term time-fractional PDE models arising in mathematical physics using local meshless method. Open Physics. 2020;18(1):1063–1072. doi: 10.1515/phys-2020-0222

[46] Ahmad H, Alam MN, Omri M. New computational results for a prototype of an excitable system. Results in Physics. 2021;28:104666. doi: 10.1016/j.rinp.2021.104666

[47] Van den Driessche P, Watmough J. Reproduction numbers and sub-threshold endemic equilibria for compartmental models of disease transmission. Mathematical biosciences. 2002;180(1-2):29–48. doi: 10.1016/S0025-5564(02)00108-6 12387915

[48] Pakistan, Key facts on HIV;. https://www.aidsdatahub.org/country-profiles/pakistan.

[49] de León UAP, Pérez ÁG, Avila-Vales E. A data driven analysis and forecast of an SEIARD epidemic model for COVID-19 in Mexico. arXiv preprint arXiv:200408288. 2020;. 10.1016/j.chaos.2020.110165 PMC7434626 32834649

[50] Life expectancy at birth, total (years)—Pakistan;. https://data.worldbank.org/indicator/SP.DYN.LE00.IN?locations=PK.

[51] Pakistan Life Expectancy 1950-2023;.

